# Social anxiety and paranoid beliefs in adolescents

**DOI:** 10.1002/jcv2.12280

**Published:** 2024-09-11

**Authors:** J. L. Kingston, B. Schlier, E. Leigh, D. Widyasari, R. P. Bentall

**Affiliations:** ^1^ Royal Holloway University of London Egham UK; ^2^ University of Hamburg Hamburg Germany; ^3^ Department of Experimental Psychology University of Oxford Oxford UK; ^4^ University of Sheffield Sheffield UK

**Keywords:** adolescents, adversity, bullying, discrimination, paranoia, social anxiety

## Abstract

**Background:**

Paranoid beliefs are common in the general adolescent population. The paranoia hierarchy suggests common social evaluative concerns may develop into persecutory thoughts via ideas of reference, a milder intermediary facet of paranoia. Socially anxious concerns and paranoid beliefs co‐occur in adolescent and adult groups, but the specifics of their association is not well understood. In a general population adolescent sample, we examined (a) whether social anxiety and paranoia can be differentiated, (b) patterns of co‐occurrence and (c) psychosocial factors that differentiate social anxiety alone versus in combination with paranoia.

**Methods:**

An online cross‐sectional survey design, recruiting UK adolescents (*n* = 604, 14–17 years), via Qualtrics. Participants were quota sampled for equal distribution on age and gender.

**Results:**

Measurement models supported a hierarchical structure, with separate but correlated general factors of paranoia and social anxiety. This model was invariant across age groups 14–15 and 16–17 years. The largest subgroup of participants with clinically significant symptoms showed elevated social anxiety plus paranoia (21%, *n* = 124), followed by high social anxiety without paranoia (14%, *n* = 84). Paranoia without social anxiety occurred the least (7% *n* = 39). Subgroup comparisons suggested social anxiety plus paranoia was characterised by exposure to threating experiences (discrimination, bullying, adverse life events in the last 12‐month), anxious attachment and high levels of distress, whereas social anxiety was more associated with feeling inferior to others, enhanced loneliness, avoidant attachment and a low sense of belonging.

**Conclusions:**

Social anxiety and paranoia are differentiable in adolescents. Paranoia commonly co‐occurs with social anxiety, especially in those with exposure to threat environments in the last 12‐month. Adolescents with social anxiety plus paranoia reported the highest levels of distress, underscoring the importance of improved understanding of this group.

**Key points**

- Paranoia and social anxiety share common features, but are differentiable in adolescents.
- Paranoia commonly co‐occurred with social anxiety; approximately half of adolescents reporting elevated social anxiety also reported elevated paranoia.
- Adolescents reporting social anxiety plus paranoia were much more likely to have experienced bullying, discrimination and adverse life events (ALEs) in the last 12‐month than those with social anxiety alone.
- The combination of social anxiety plus paranoia was associated with the high levels of distress, low perceived school membership and anxious attachments to significant others
- Findings highlight the need for better identification, understanding, and treatment for adolescents with social anxiety plus paranoia.

## INTRODUCTION

Current models of paranoia focus almost exclusively on research from adults (18+ years) yet fears about others' harmful intentions emerge earlier in development (Bird et al., 2021; Kingston et al., 2022; Wong et al., 2014). Unsurprisingly, these fears are often grounded in exposure to threatening life events, with paranoia being more common in adolescents reporting bullying, victimisation, and adverse life events (ALEs) (Bird et al., 2022; Kingston et al., 2023). In general population UK adolescents, paranoia is associated with poor peer relationships and depression (Bird et al., 2019), and reduced self‐esteem and well‐being over time (Kingston et al., 2022). Paranoia is an under‐recognised aspect of adolescent mental health and the absence of a conceptual framework for how paranoid beliefs develop during adolescence is a significant gap in current literature.

Although historically viewed as a sign of severe mental illness, paranoia is increasingly understood within the context of anxiety; that is, an anxious fear about others' harmful intentions (Freeman et al., 2008). Social evaluative concerns (e.g., interpersonal worry, fears of rejection), which are thought to underpin paranoia (Freeman et al., 2005), are common during adolescence, where self and identity are developing, belonging within a social network is highly valued, and sensitivity to criticism and rejection is paramount (Marston et al., 2010). According to the paranoia hierarchy (Freeman et al., 2005), common social evaluative concerns will for some develop into paranoid beliefs, of which two types are identified. *Ideas of reference,* a milder intermediary facet of paranoia, describes the tendency to detect personal significance in otherwise neutral events. For some, this may escalate into *persecutory thoughts* of increasing severity, with persecutory delusions being the most sever. However, the extent to which this model applies to adolescent paranoia has not previously been tested, and the factors (e.g., life experiences, psychological mechanisms) affecting transitions from social evaluative concerns to paranoid beliefs have not been articulated or examined.

In adult clinical and general population samples, paranoia and social anxiety commonly co‐occur (e.g., Michail & Birchwood, 2009; Pallanti et al., 2013; Rietdijk, Van Os, de Graaf, Delespaul & van der Gaag, 2009). For example, in adults with either first episode psychosis or social anxiety disorder without psychosis, Michail and Birchwood (2009) reported that multiple developmental pathways likely existed (e.g., social anxiety preceding, triggering and maintaining paranoia; both developing concurrently during the early stages of psychosis and following similar yet separable courses; social anxiety developing as consequence of paranoia). However, by recruiting a group with already established diagnoses, as well as using cross‐sectional design, the extent to which findings reflect the *development* of these difficulties is limited. Using NEMISIS cross‐sectional and longitudinal data in a general population adult Dutch sample, Rietdjik et al. (2009) reported that 19% of individuals with subclinical paranoid symptoms reported social anxiety disorder and 23% of those with social anxiety disorder reported subclinical paranoid symptoms. The odds of having social anxiety disorder alongside paranoid symptoms increased substantially with the increasing number of paranoid beliefs endorsed. Furthermore, in the longitudinal data, subclinical paranoid symptoms were prospectively associate with onset of social anxiety disorder 1–3 years later, with no evidence for the converse. However, authors excluded participants whose symptoms began before 18 years of age, whereas in most cases these difficulties start during adolescents (Kessler et al., 2005).

Only two studies have investigated social anxiety and paranoia in adolescents. Firstly, Pisano et al. (2016) reported elevated paranoia in adolescents with social anxiety disorder relative to age and sex‐matched adolescents without mental health problems. Secondly, a longitudinal general population study in 17–28‐year‐olds (Schutters et al., 2012) reported that paranoia and social anxiety disorder co‐occurred more than would be expected by chance. However, co‐occurrence was low (2.2%) and less common than paranoia (13.5%) or social anxiety disorder (7.1%) alone. Authors concluded that despite commonalities, the two diverged more than they converged. However, paranoia was assessed using four items of severe and rarely endorsed content (e.g., being spied on, being secretly tested). The sensitivity and validity of this method of measuring paranoid concerns in adolescents is unclear, and the absence of items capturing less severe paranoid concerns may conceivably have led to an underestimation of co‐occurrence with social anxiety.

The current study represents the first step in examining the hypothesis that adolescents sensitive to social evaluative concerns who have experienced threatening life events will additionally report paranoid beliefs. First, we tested whether social anxiety and paranoia can be differentiated in adolescents by comparing measurement models in which the facets of these two constructs are factors of the same overarching construct (hierarchically structured) versus separable constructs. For the model with the highest fit indices, we additionally assessed measurement‐invariance across age groups as a necessary pre‐requisite for subsequent full sample analyses. Second, we examined patterns of co‐occurrence for elevated/above threshold levels of all three. We reasoned that if paranoia builds on socially anxious concerns, via a less clinical form of paranoia (i.e., ideas of reference), this would be reflected by: (a) most adolescents scoring below threshold on all three, followed by (b) elevated social anxiety only, (c) elevated social anxiety plus paranoia. Within (c), we hypothesized that the largest group would be (c1) social anxiety plus ideas of reference, followed by (c2) all three (social anxiety, ideas of reference, and persecutory thoughts) co‐occurring, with (c3) social anxiety and persecutory thoughts being the rarest combination. Similarly, the remaining category (d) paranoia without social anxiety was hypothesized to be the rarest/not observed. Third, we tested whether adolescents with social anxiety plus paranoia versus social anxiety alone, paranoia alone, or those reporting neither social anxiety or paranoia showed differences across a set of psychosocial variables. We investigated exposure to threatening life events (bullying, victimisation, ALEs), interpersonal functioning (attachment in close relationships, loneliness, social comparison, and sense of belonging) and distress. We anticipated that exposure to threat would characterise those with social anxiety plus paranoia versus social anxiety alone but had no a priori predictions for the remaining variables.

## METHODS

### Design and participants

A cross‐sectional survey collecting self‐report data from UK dwelling adolescents (*n* = 604). Adolescents were recruited via Qualtrics online participant recruitment service. Parents of adolescents registered with Qualtrics panels were contacted for permission to invite their adolescent child to take part. Quota sampling ensured a 50:50 gender split and a 50:50 distribution across age groups 14–15 and 16–17 years. The survey link was opened by *n* = 1362 parents. Of these, *n* = 705 parents and adolescents provided consent. Only those completing all questionnaires were retained, resulting in *n* = 101 being excluded for discontinuing before the end.^1^ All participants in the final dataset met pre‐determined data quality criteria (completing below a speed threshold, UK geographical location, sensible responses to open text boxes, passing two randomly distributed attention checks).

### Measures

Descriptive and socio‐demographic variables: age, gender, household income, country of birth and current self‐reported mental health diagnosis (yes/no).

*The Revised Green et al., Paranoid Thoughts Scale* (RGPTS; Freeman et al., 2021), an 18‐item measure of paranoia with subscales assessing ideas of reference (8‐items, e.g., “I spent time thinking about friends gossipping about me”, current sample: *α* = 0.94) and persecutory thoughts (10‐items, e.g., “I was sure someone wanted to hurt me”, current sample: *α* = 0.96). Items are rated on a 5‐point scale (*0–not at all* to *4–totally*) with high scores (range 0–40) indicate high paranoid beliefs. For persecutory thoughts, we used the validated cut‐off of ≥18 (indicating more than moderate (11–17) and at least severe levels (18–27) of persecutory thoughts; Freeman et al., 2021). In the absence of a similarly validated cut‐off for ideas of reference, we used the lower end of the “severe levels of ideas of reference” category (≥21) to parallel the cut‐off for persecutory thoughts. Psychometric assessment of R‐GPTS has supported its use in this age group (Schlier et al., 2024).

*Social Anxiety Scale for Adolescents* (SAS‐A; La Greca & Lopez, 1998), an 18‐item measure with items rated from 1‐*not at all characteristic of me* to 5‐*extremely characteristic of me,* forming three subscales: Fear of Negative Evaluation (8‐items, e.g., “I worry about what others think of me”, current sample: *α* = 0.96), Social Avoidance and Distress in New Situations (6‐items, e.g., “I get nervous when I meet new people”, current sample: *α* = 0.95) and more pervasive Social Distress (4‐items, e.g., “I feel shy even with peers I know well”, current sample: *α* = 0.89). High levels of social anxiety were defined as SAS‐A total score ≥50 (La Greca, 1999).

*The ALEs Scale* (Tiet et al., 1998), a 25‐item measure of negative life events in the last 12‐month (e.g., a close friend was seriously sick/injured). Participants rate (yes/no), with “yes” followed by questions assessing how negative the experience was (mostly good, mostly bad, NA, don't know) and how much it affected them (not at all – a lot). Using established scoring procedures, a total adverse event score was computed by summing the number of “mostly bad” events that affected them at least “a little”. Higher scores (range 0–25) indicate more ALEs. A probability sample of 9–17‐year‐olds in the USA reported a mean 12‐month frequency of 1.97^2^.

*The Brief Self‐Report Measure of Adolescent Bullying–Victimisation* (Murray et al., 2021), a 5‐item measure of bullying in the last 12‐month (current sample: *α* = 0.80). Participants read a brief introduction, followed by 5 examples of being bullied (e.g., purposefully ignored; hit, bitten, kicked). Adolescents estimate frequency (never, 1–2 times, 3–10 times, about once a month, about once a week, (almost) every day) in the last 12‐month. Higher scores (range 0–25) indicate greater incidence.

*The Everyday Discrimination Scale* (Williams et al., 1997), a 5‐item measure of the frequency of discriminatory experiences (1‐*never* to 5‐*almost everyday*) without reference to any particular domain. Items range from mild (treated with less courtesy) to more chronic experiences (threatened or harassed). High scores (range 6–30) indicate higher discrimination (current sample: *α* = 0.80).

*The Depression Anxiety Stress‐short form* (DASS‐21; Henry & Crawford, 2005) measures depression, anxiety and tension/stress (7 items per subscale) over the previous week (0‐*Did not apply to me at all* to 3‐*Applied to me very much/most of the time*). Authors reported excellent internal consistency (*α* = 0.93) with comparable results in the current sample (*α* = 0.97). The total score (range: 0–63) as well as subscale scores for depression, anxiety, and stress (range: 0–21, respectively) were calculated.

*The Social Comparison Scale* (SCS; Allan & Gilbert, 1995) includes 11‐items (e.g., inferior ‐superior, outsider‐insider) which participants rate from one (e.g., inferior) to 10 (e.g., superior) using the response stem “in relation to others I feel…”. Items are rated over the last four weeks and higher scores thus indicate a more positive view of oneself in relation to others (current sample: *α* = 0.93).

*The Experiences in Close Relationships ‐ Revised – General Short Form* (Wilkinson, 2011) is a 20‐item measure assessing general relationship attachment (anxious and avoidant, 10‐items each) in adolescents and young adults. Items are rated using a 5‐point scale from 1‐*strongly agree* to 5‐*strongly disagree.* Good reliability (*α* = 0.88) and validity has been reported by the authors (current sample: *α* = 0.92).

*The UCLA Loneliness scale* (Wongpakaran et al., 2020) assessed loneliness via six items, with each rated from 1‐hardly ever or never to 3‐often. The UCLA has shown good psychometric properties in adolescent samples (current sample: *α* = 0.95).

*The Psychological Sense of School Membership Scale* (Ye & Wallace, 2014), an 18‐item measure of school belonging. Items are rated on a 5‐point scale from 1‐*not at all true* to 5‐*completely true*) and authors reported good reliability and validity in adolescent samples (current sample: *α* = 0.94).

Similar to McIntyre et al. (2018) we adapted Doosje et al.’s (1995) measure of ingroup identification to assess how much participants felt “strong group ties”, “belongingness” and “identification” with a range of adolescent social domains (e.g., peers, class, clubs) using a scale of 0‐“not at all” to 3‐“very much”. Total belongingness scores ranged from 0 (low) to 72 (high).

### Data analysis plan

Data was analysed using SPSS 26 and R 4.2.2. First, we compared measurement models with or without differentiation between paranoia and social anxiety, using confirmatory factor analyses on the R‐GPTS and SAS‐A and testing four different models. Model 1 (“one general factor”) had all R‐GPTS and SAS‐A items load on one common factor. Model 2 (“two general factors”) had R‐GPTS items load on a first factor and SAS‐A items on second one, while allowing general factors to correlate. Model 3 (“hierarchic with one second order factor”) had items allocated to the respective subscales of the R‐GPTS (ideas of reference, persecutory thought) and the SAS‐A (fear of negative evaluation, avoidance in new situations, general social avoidance), which then both loaded on one general second order factor. Model 4 (“hierarchic with two second order factors”) used the same first order factors but had R‐GPTS and SAS‐A subscales load on separate, correlated second‐order factors representing paranoia and social anxiety, respectively. Absolute model fit was evaluated using CFI (sufficient fit: CFI>0.90, good fit: CFI>0.95), RMSEA (sufficient fit: RMSEA<0.08, good fit: RMSEA<0.04), and SRMR (sufficient fit: SRMR<0.08). Additionally, model fit in comparison to the other models was evaluated using the AIC and the BIC (Hopwood & Donnellan, 2010; Hu & Bentler, 1999). Measurement invariance was tested between the age‐subsamples 14–15 and 16–17, using the aforementioned cutoffs for CFI, RMSEA, and SRMR for determining configural invariance (equal factor structure), and specific cut‐offs for the difference in indices compared to the model at the prior stage of invariance analysis (Chen, 2007) for metric‐invariance (equal structure and loadings; ΔCFI>−0.010, ΔRMSEA<0.015, and ΔSRMR<0.030), scalar invariance (equal structure, loadings, and intercept; ΔCFI > −0.010, ΔRMSEA <0.015, and ΔSRMR <0.015), and residual invariance equal structure, loadings, intercepts, and residuals; ΔCFI > −0.010, ΔRMSEA <0.015, and ΔSRMR <0.015)

Secondly, patterns of co‐occurrence for over‐threshold scores on social anxiety, ideas of reference, and persecutory thought were examined using the pre‐existing cut‐offs for the SAS‐A and R‐GPTS (see measures). We then summarized the co‐occurrence for high levels of social anxiety, ideas of reference, and persecutory thoughts and visualized the overlap between prevalence's using the R‐package eulerr (Larsson & Gustafsson, 2018).

Finally, to test for differences in psychosocial risk factors for social anxiety alone, versus ideas of reference/persecutory thought alone, versus social anxiety with ideas of reference/persecutory thought, we converted cut‐off scores into a single variable with four levels: (a) “both low” ‐ low scores on social anxiety and paranoia (ideas of reference and persecutory thought), (b) “social anxiety high” ‐ participants who scored above the cutoff (SAS‐A‐total) for social anxiety only, (c) “paranoia high” – participants with least one paranoia score above cutoff (i.e. persecutory thoughts, ideas of reference, or both), and (d) “both high” ‐ participants with social anxiety (SAS‐A‐total) and at least one paranoia score above cutoff. The resulting Group factor was used as an independent variable in 10 ANOVAs with one of the psychosocial risk factors (*threat experiences*: bullying, discrimination, total ALEs; *interpersonal relationships and belonging*: anxious/avoidant attachment, school membership, total belonging, loneliness, social comparison; and *distress*: DASS‐21) as dependent variables. We tested for overall between Group differences, followed by Bonferroni corrected post hoc tests.

## RESULTS

### Participant demographics and characteristics

On average, participants were 15.48 years old (SD = 1.06). Two hundred and ninety‐nine participants (49.5%) identified as female, 298 (49.3%) male, with the remaining identifying as trans‐female (0.2%), trans‐male (0.7%) or gender queer (0.3%). Most participants were white (87.4%). Most reported an average (61.3%) or below average (24.3%) socio‐economic status. Overall, 20.2% of the sample reported having one or more self‐reported mental health diagnosis (most common: anxiety (13.2%), depression (3.1%), autism spectrum disorders (3.1%), and ADHD (3.0%). Forty‐one participants (6.8%) reported taking medication for this (see Table 1 for full sample details).

**TABLE 1:** Sociodemographic data on the study sample.

	Full sample	Age group 14–15	Age group 16–17
N	604	304	300
Age (M; SD)	15.48; 1.06	14.54; 0.50	16.42; 0.49
Gender			
Female	299 (49.5%)	151 (49.7%)	148 (49.3%)
Male	298 (49.3%)	150 (49.3%)	148 (49.3%)
Trans female	1 (0.2%)	0	1 (0.3%)
Trans male	4 (0.7%)	1 (0.3%)	3 (1.0%)
Gender queer	2 (0.3%)	2 (0.7%)	0
Ethnicity			
White	528 (87.4%)	266 (87.5%)	262 (87.3%)
Asian	21 (3.5%)	9 (3.0%)	12 (4.0%)
Black	11 (1.8%)	7 (2.3%)	4 (1.3%)
Mixed	38 (6.3%)	20 (6.6%)	18 (6.0%)
Other	6 (1.0%)	2 (0.7%)	4 (1.3%)
Socio‐economic status			
Below average	147 (24.3%)	78 (25.7%)	69 (23.0%)
Average	370 (61.3%	183 (60.2%)	187 (62.3%)
Above average	53 (8.8%)	26 (8.6%)	27 (9.0%)
Unknown	34 (5.6%)	17 (5.6%)	17 (5.7%)
Mental health problems			
None	482 (79.8%)	240 (78.9%)	242 (80.7)
Any problems	122 (20.2%)	64 (21.1%)	58 (19.3%)
Anxiety	80 (13.2%)	42 (13.8%)	38 (12.7%)
Depression	19 (3.1%)	8 (2.6%)	11 (3.7%)
Autism spectrum disorder	19 (3.1%)	11 (3.6%)	8 (2.7%)
ADHD	18 (3.0%)	12 (3.9%)	6 (2.0%)
Other	17 (2.8%)	7 (2.3%)	10 (3.3%)
Taking medication for mental health problems	41 (6.8%)	18 (5.9%)	23 (7.7%)

### Factor structure of social anxiety and paranoia

Descriptive statistics and response distribution for all R‐GPTS and SAS‐A items are provided in the supplementary materials (Supporting Information S1: Table S1). CFA of the R‐GPTS and SAS‐A yielded insufficient model fit for any model without a hierarchical structure. Among the models with hierarchical structure, the model with two second order factors for paranoia and social anxiety yielded the best model fit and was the only model with sufficient model fit according to all fit indices (robust CFI = 0.917, robust RMSEA = 0.074, SRMR = 0.077; for a full list of fit indices, see Table 2). Whereas social anxiety and paranoia were separate constructs, they remained highly correlated (standardized covariance: 0.759; see supplementary materials Figure S1 for a full visual presentation of the models and Supporting Information S1: Table S2 for all item loadings). Subsequent invariance analyses showed no indication of non‐invariance of the model with two second order factors for paranoia and social anxiety (see Table 2).

**TABLE 2:** Results of confirmatory factor analyses of the R‐GPTS and the SAS‐A in the full sample (upper half) versus Age specific samples (lower half).

Model	CFI robust (>0.90)	RMSEA robust (<0.08)	SRMR (<0.08)	AIC		BIC
Model 1: One general factor	0.681	0.145	0.106	55,662		55,979
Model 2: Two general factors (R‐GPTS vs. social anxiety)	0.811	0.112	**0.076**	52,575		52,896
Model 3: Hierarchical with one second order factor (R‐GPTS/SAS‐A subscales as first order factors)	**0.906**	**0.079**	0.086	50,326		50,665
Model 4: Hierarchical with two second order factors (paranoia vs. social anxiety) with R‐GPTS/SAS‐A subscales as first order factors	**0.917**	**0.074**	**0.077**	50,059		50,402

Model 4 – Age invariance analysis (14–15 vs. 16–17)	CFI robust (>0.90)	RMSEA robust (<0.08)	SRMR (<0.08)	ΔCFI	ΔRMSEA	ΔSRMR
Configural invariance	**0.916**	**0.075**	**0.078**			
Metric invariance (loadings)	**0.916**	**0.074**	**0.081**	**0.000**	**−0.001**	**+0.003**
Scalar invariance (loading + Intercepts)	**0.916**	**0.074**	**0.081**	**0.000**	**0.000**	**0.000**
Residual invariance (loadings + intercepts + residual variance)	**0.912**	**0.074**	**0.081**	**−0.004**	**0.000**	**0.000**

*Note*: Fit indices printed in bold denote values above (CFI)/below (RMSEA, SRMR) the respective threshold for sufficient model fit or differences sufficiently small to assume invariance.

### Prevalence and overlap of social anxiety and paranoia

Summarizing SAS‐A and R‐GPTS using the respective cutoffs, most participants (59%, *n* = 357) did not show any elevated levels of social anxiety, ideas of reference or persecutory thoughts. Thirty‐four percent (*n* = 208) reported high levels of social anxiety, 16.1% (*n* = 97) with high levels of ideas of reference, and 22.7% (*n* = 137) with high levels of persecutory thoughts.

**FIGURE 1:**
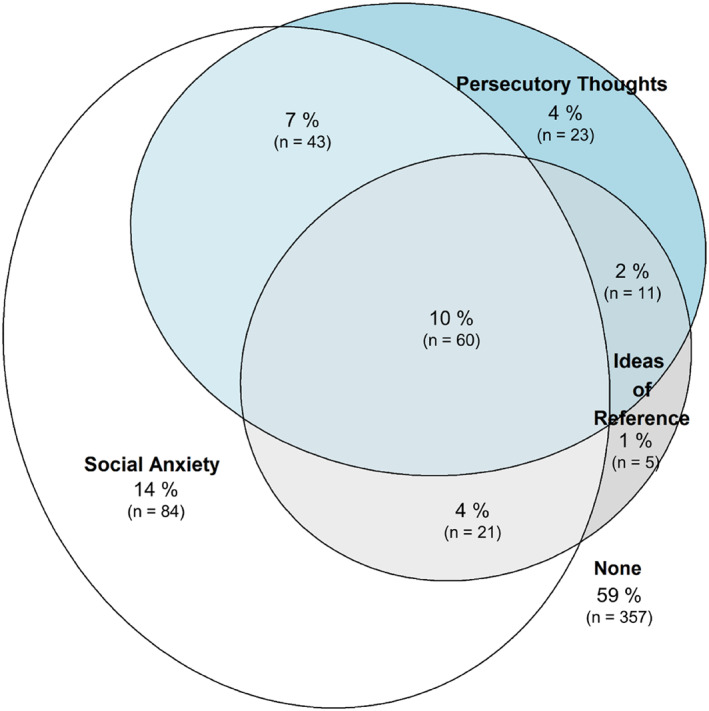
Venn‐diagram of overlap between prevalence of social anxiety, ideas of reference, and persecutory thoughts.

Figure 1 summarises the overlap between high levels of social anxiety, ideas of reference, and persecutory thoughts. Contrary to expectation, the largest subgroup of participants with elevated symptoms showed a combination of high social anxiety plus paranoid beliefs (21%, *n* = 124). Further, within this group, the combination of all three was most common (10%, *n* = 60), followed by social anxiety and persecutory thought (7%, *n* = 43), and social anxiety and ideas of reference (4%, *n* = 21) being the rarest. Following this, the high social anxiety only group were the third largest group, which was contrary to our initial hypotheses (14%, *n* = 84). Participants with high levels of ideas of reference and/or persecutory thoughts but without high social anxiety were considerably less prevalent (6%, *n* = 39).

### Differences in psychosocial risk factor between cut‐off groups

Table 3 reports sociodemographic information for participants across groups (both low vs. high social anxiety (only) versus high paranoia (only) versus both high). Social anxiety and paranoia were higher in girls than boys. Age also significantly differed across groups, with those reporting elevated paranoia (alone or alongside social anxiety) being younger. Uncorrected post‐hoc tests for demographic variables (for which no hypotheses existed) showed that participants with high social anxiety and paranoia were significantly younger than participants without any elevated symptom levels (*T* = −2.55, *p* = 0.012, *d* = −0.26) or with social anxiety only (*T* = −2.75, *p* = 0.006, *d* = −0.39). Participants with high paranoia (only) were significantly younger than participants with high social anxiety only (*T* = −2.26, *p* = 0.024, *d* = −0.44). Self‐reported mental health diagnoses, in particular anxiety and depression, were more common in those with elevated social anxiety, paranoia and especially both combined.

**TABLE 3:** Sociodemographic data on the study sample by group.

	Both low	SA high	Paranoia high^a^	Both high	Test for differences
N	357	84	39	124	
Age (M; SD)	15.53; 1.07	15.67; 1.06	15.21; 0.95	15.26; 1.05	F(3, 600) = 3.91, *p* = 0.009, *η* ^2^ = 0.019
Gender					
Female	153 (42.9%)	48 (57.1%)	24 (61.5%)	74 (59.7%)	
Male	202 (56.6%)	35 (41.7%)	15 (38.5%)	46 (41.7%)	χ^2^(12) = 27.875, *p* = 0.006
Trans female	0	0	0	1 (0.8%)	
Trans male	2 (0.6%)	0	0	2 (1.6%)	
Gender queer	0	1 (1.2%)	0	1 (0.8%)	
Ethnicity					
White	302 (84.6%)	77 (91.7%)	36 (92.3%)	113 (91.1%)	χ^2^(12) = 10.84, *p* = 0.543
Asian	17 (4.8%)	1 (1.2%)	0	3 (2.4%)	
Black	7 (2.0%	1 (1.2%)	1 (2.6%)	2 (1.6%)	
Mixed	25 (7.0%)	5 (6.0%)	2 (5.1%)	6 (4.8%)	
Other	6 (1.7%)	0	0	0	
Socio‐economic status					
Below average	70 (19.6%)	27 (32.1%)	12 (30.6%)	38 (30.6%)	χ^2^(9) = 15.91, *p* = 0.069
Average	233 (65.3%)	47 (56.0%)	24 (61.5%)	66 (53.2%)	
Above average	35 (9.8%)	4 (4.8%)	3 (7.7%)	11 (8.9%)	
Unknown	19 (5.3%)	6 (7.1%)	0	9 (7.3%)	
MH problems					
None	316 (88.5%)	59 (70.2%)	29 (74.4%)	79 (63.7%)	χ^2^(3) = 42.47, *p* < 0.001
Any problems	41 (11.5%)	25 (29.8%)	10 (25.6%)	45 (36.3%)	
Anxiety	23 (6.4%)	19 (6.4%)	5 (12.8%)	33 (26.6%)	χ^2^(3) = 40.09, *p* < 0.001
Depression	2 (0.6%)	4 (4.8%)	1 (2.6%)	11 (8.9%)	χ^2^(3) = 23.06, *p* < 0.001
ASD	7 (2.0%)	4 (4.8%)	1 (2.6%)	7 (5.6%)	χ^2^(3) = 4.95, *p* = 0.175
ADHD	8 (2.2%)	3 (3.6%)	3 (7.7%)	5 (4.0%)	χ^2^(3) = 3.98, *p* = 0.264
Other	7 (2.0%)	5 (6.0%)	2 (5.1%)	4 (3.2%)	χ^2^(3) = 4.50, *p* = 0.213
Taking medication for MH problems	13 (3.6%)	7 (8.3%)	4 (10.3%)	17 (13.7%)	χ^2^(3) = 16.03, *p* = 0.001

Abbreviations: MH, mental health; ASD, autism spectrum disorder.

^a^ Ideas of reference or of persecution.

**FIGURE 2:**
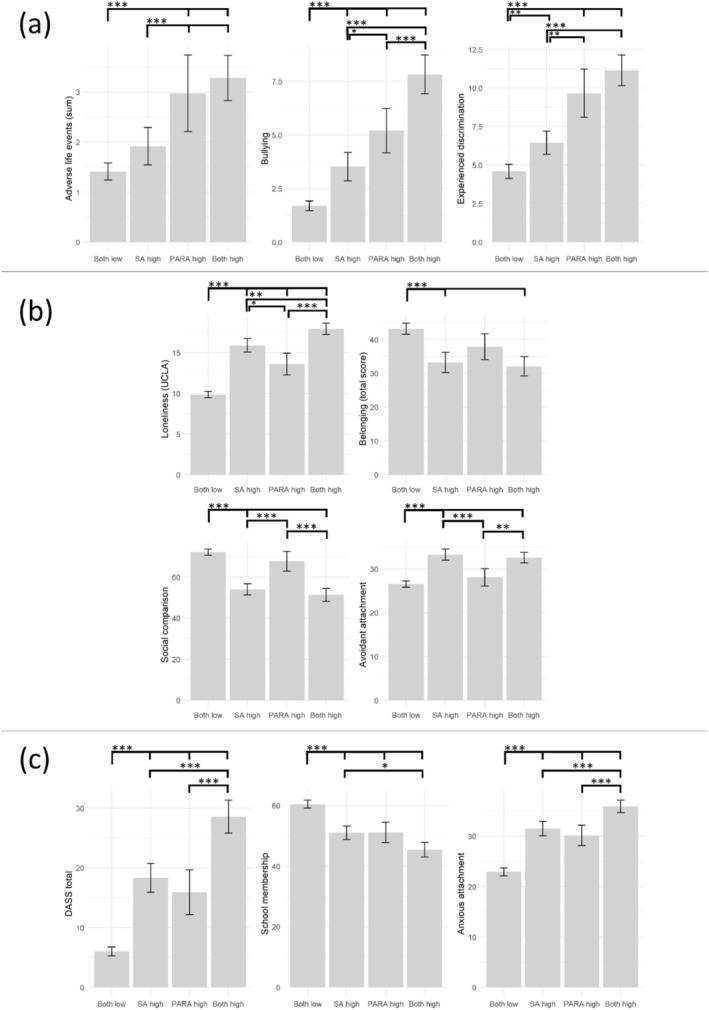
Psychosocial risk factor showing (A) more severe scores in cut‐off groups with high paranoia (B) more severe scores in cut‐off groups with high social anxiety and (C) comparable differences for above cut‐off paranoia and above cut‐off social anxiety (mean values with uncorrected 95% confidence interval. Significant post‐hoc between group comparisons with Bonferroni‐corrections are highlighted with annoted bars; *** ‐ *p* < 0.001, ** ‐ *p* < 0.01, * ‐ *p* < 0.05).

All one‐way ANOVAs with cut‐off group as the independent variable and one of the ten psychosocial risk factors as dependent variable showed a significant main effect (all *p* < 0.001, see Supporting Information S1: Table S3). Bonferroni‐corrected post‐hoc tests (see Supporting Information S1: Table S4) identified three clusters of psychosocial risk factors, based on their distinct pattern of between group differences (see Figure 2; descriptive values and Supplement Tables S5 and S6; intercorrelation of measures).

The first category of ALEs, bullying, and discrimination showed that the high paranoia and both high groups had significantly more negative mean values (e.g., more discrimination) than the both low and the social anxiety high group. For bullying and experiences of discrimination, the social anxiety high group showed significantly elevated levels compared to the both low group, but significantly lower levels than the paranoia and both high groups. For ALEs, there were no differences between the both low and social anxiety high groups.

The second category included loneliness, overall feeling of belonging, social comparison, and avoidant attachment. For these risk factors, the high social anxiety and both high groups showed the most extreme mean values (e.g., more loneliness, less feeling of belonging), significantly differing from the both low group. The paranoia high group either showed intermediary mean values significantly higher than the both low group, but lower than the social anxiety/both high group (Loneliness, Belonging) or did not differ from the both low group (social comparison, avoidant attachment).

Regarding the remaining risk factors, general distress (DASS‐21), school membership, and anxious attachment, there was a pattern of the both low group showing the most favourable mean scores and the both high group showing the most unfavourable mean scores. However, no clear distinctions between the social anxiety and paranoia could be derived from the post‐hoc comparisons.

## DISCUSSION

This study had three main aims: firstly, we used measurement models to examine whether social anxiety and paranoia can be differentiated in adolescents; secondly, we examined the co‐occurrence of above threshold scores on social anxiety and facets of paranoia (i.e., ideas of reference and of persecution) to examine whether patterns conformed to those anticipated by the paranoia hierarchy; thirdly, we compared groups of adolescents with different patterns of co‐occurrence (low on both, high on both, high social anxiety and low paranoia, and high paranoia low social anxiety) to test whether exposure to threatening life events characterised participants with social anxiety plus paranoia versus social anxiety alone and whether groups different on anxious/avoidant attachment, school membership, belongingness, loneliness, social comparison and general distress.

Our results offer new and important findings. First, social anxiety and paranoia were differentiable. This was indicated not only in the measurement models, but also in the differences between groups defined by their levels of social anxiety and paranoia. Here, distinct profiles on key psychological, social and emotional variables suggested important points of differentiation.

Secondly, approximately 50% of adolescents reporting elevated levels of social anxiety also reported significant paranoid concerns (i.e., severe range for persecutory thoughts, ideas of reference or both). Conversely, paranoia without social anxiety was considerably less common. Overall, these findings are consistent with the high rates of co‐occurrence between social anxiety, paranoia and psychosis in adult general and clinical populations (Michail & Birchwood, 2009; Rietdijk et al., 2009) and with reports of elevated paranoia in adolescents with social anxiety (Pisano et al., 2016; Schutters et al., 2012). However, the findings are unique in their focus on younger adolescents and in examining co‐occurrence in the general population, rather than specific diagnostic groups. As such, they have broader implications for conceptualising these experiences in general population adolescents. The overall pattern of paranoia being relatively common in adolescents reporting elevated social anxiety (i.e., in 50% of cases), yet relatively rare in the absence of elevated social anxiety, is consistent with the premise of the paranoia hierarchy (i.e., that paranoia builds on socially anxious concerns). However, instances of paranoia without social anxiety are at odds with the hierarchy perspective, suggesting a route into paranoia that is not via social anxiety. This subgroup reported highest exposure to threat (discrimination, bullying, ALEs), whilst also reporting a greater sense of belonging, less loneliness and inferiority concerns, and less avoidance in relationships than the high social anxiety and both‐high group. It is possible that this is a group of adolescents with fears about particular others, perhaps arising from specific adverse experiences, but who may also have protective relationships. The small sample size limits firm conclusions, yet this represent an interesting subgroup for future investigation.

Thirdly, adolescents reporting paranoia (ideas of reference and/or persecutory thoughts) alongside elevated social anxiety were significantly more likely to have been exposed to bullying, discrimination and ALEs in the last 12‐month than the social anxiety only group. Interestingly, similar patterns were observed across all three measures of threat exposure, suggesting this effect is not specific to any one type of experience. Consistent with our a priori hypothesis, this could indicate that exposure to high threat and/or adverse environments is an important factor for determining adolescents who experience social anxiety alone versus in combination with paranoia. One possible interpretation is that threatening life events influence the transitions from socially anxious concerns to paranoia; however, the temporal nature of this hypothesis could not be tested, nor other interpretations ruled out. For example, it is equally possible that in the high‐high group, exposure to threatening life events gave rise to social anxiety and paranoia concurrently rather than sequentially. Future research should investigate the temporal association between social anxiety and paranoia using prospective observational studies. Examining the psychosocial mechanisms/causal pathways that explain this link (e.g., fears about being vulnerable to harm from others, avoidance behaviours) can help inform treatment targets. Furthermore, whether the overlap between social anxiety and paranoia is unique to these two variables, or reflects a broader overlap between paranoia and anxiety and/or distress is an important future direction.

Finally, adolescents experiencing elevated social anxiety and paranoia have typically not been the focus of prior research. Our data identified this group as experiencing the highest level of distress, alongside low perceived school membership and anxious attachments to significant others. In the adult literature, individuals at clinical high risk for psychosis with concurrent social anxiety have lower quality of life and self‐esteem than those at clinical high risk for psychosis without social anxiety (Room et al., 2012). Likewise, adolescents with anxiety/depression and co‐occurring psychotic symptoms had poorer long‐term prognosis for recovery compared to those with anxiety/depression only (Wigman et al., 2012). Early detection of individuals on a possible trajectory for long term and hard to treat mental heath conditions is of the utmost importance and our data suggest that measuring paranoia alongside social anxiety *could* be means of detecting cases of concern, although further research is needed.

Regarding the two facets of paranoia, that is, milder ideas of reference that bridge the path from social concerns/social anxiety to persecutory thoughts of increasing severity (Freeman et al., 2005), some of our findings were counter to expectation: On the one hand we replicated the finding that paranoia includes the distinguishable facets of ideas of reference and persecutory thoughts in adolescents. On the other hand, instances of social anxiety plus persecutory thoughts (7%, *n* = 43) were almost twice as common as social anxiety plus ideas of reference but not persecution (4%, *n* = 21). Likewise, instances of all three in combination (10%, *n* = 60), were more common than other combinations. Taken together, this data suggests that, in adolescents, ideas of reference are not an intermediary step between social anxiety and persecutory thoughts, as proposed in the paranoia hierarchy. Only a small number of participants experienced ideas of reference in isolation (1%, *n* = 5), without social anxiety (3%, *n* = 16), or without persecutory thoughts (4%, *n* = 26). Rather, over threshold rates of ideas of reference mostly came hand‐in‐hand with persecutory thoughts, perhaps suggesting that ideas of reference are a feature of paranoid beliefs rather than a pathway to paranoid beliefs. Further examination in prospective cohorts is required.

Findings should be considered in light of several limitations. Findings are preliminary and based on cross‐sectional methods. Longitudinal assessment of the links between social anxiety and paranoid beliefs are an important next step. It needs noting that self‐report measures of related constructs incur the risk of inflated correlation due to similar item content. In case of R‐GPTS and SAS‐A, some content overlap occurred for ideas of reference (R‐GPTS) and fear of negative evaluation (SAS‐A) items (see Supporting Information S1: Table S1 for item content lists). Specifically, two items of ideas on the reference scale (“people definitely laughed at me behind my back”, “people talking about me behind my back upset me”) and fear of negative evaluation scale (“I feel that others make fun of me”, “I feel that my peers talk about me behind my back”) tap into similar concepts and/or wording. While this might inflate the overlap between ideas of reference and social anxiety, this needs to be considered in light of the total pattern of overlap (Figure 1): most of the overlap between paranoia and social anxiety relates to people scoring high on persecutory thought, or persecutory thought and ideas of reference, but not ideas of reference only. Thus, the total overlap between social anxiety and paranoia is unlikely to be inflated by content overlap pertaining to ideas of reference and social anxiety.

Relatedly, the use of self‐report measures introduces issues of common method variance. Also, measures are not diagnostic and future research should seek to replicate findings using more objective assessments (e.g., interviews). Furthermore, in the absence of a pre‐established cut‐off for the ideas of reference subscale, we established our own based on pre‐established thresholds for severity. The RGPTS was also established based on adults and validation with adolescents is in its early stages (Schlier et al., 2024). Whilst the sample captured an even split for gender and age categories, the sample predominantly identified as White and are from a high‐income country. This significantly limits inferences to other, especially minoritised, groups.

Notwithstanding these limitations, the findings have important Implications. Our findings add to an emerging literature on the prevalence and impact of paranoid concerns during adolescence and underscore the importance of measuring paranoia in adolescents. Our findings advocate for measuring paranoia in adolescents with social anxiety. We found important emotional, psychological and social difference between adolescents with social anxiety with and without paranoia, which suggests that assessing paranoid beliefs in adolescents with social anxiety could assist clinicians in better understanding their treatment needs. This is especially important because research suggests that adolescents may not raise paranoid concerns with others despite having, and feeling distressed by, these concerns (Bird et al., 2022). Proactively measuring paranoia could help overcome these barriers. Whether or not paranoia alongside social anxiety impacts on the successful delivery of psychological interventions is an important area for future research.

In conclusion, social anxiety and paranoia can be differentiated in adolescents, but nonetheless have high rates of co‐occurrence. Approximately half of adolescents with elevated social anxiety report elevated paranoia, signalling that this is an important – yet typically unassessed ‐ aspect of social anxiety in adolescents. Furthermore, those reporting social anxiety plus paranoia reported the highest levels of distress, underscoring the importance of improved understanding of this group.

## AUTHOR CONTRIBUTIONS

**J. L. Kingston**: Conceptualization; data curation; funding acquisition; investigation; methodology; project administration; resources; software; supervision; validation; visualization; writing – original draft. **B. Schlier**: Conceptualization; formal analysis; visualization; writing – review & editing. **E. Leigh**: Conceptualization; visualization; writing – review & editing. **D. Widyasari**: Conceptualization; writing – review & editing. **R. P. Bentall**: Conceptualization; methodology; supervision; visualization; writing – review & editing.

## CONFLICT OF INTEREST STATEMENT

The Authors have declared that there are no conflicts of interest in relation to the subject of this study.

## Ethical considerations

Consent and ascent was obtained from all participants.

## Supporting information

Supporting Information S1

## Data Availability

Data openly available in a public repository that issues datasets with DOIs. The OSF link is osf.io/b46vs.

## ENDNOTES

^1^

*N* = 31 consented but terminated during the initial demographics phase; *n* = 70 dropped out during the questionnaire completion phase.

^2^
Internal consistency is not reported for the Adverse Live Events Scale because it does not constitute a measure of a unified construct, but a list of theoretically uncorrelated events. Hence, high internal consistency cannot be used as a fitting quality index for this questionnaire.
