# Supplementary material for: Social anxiety and paranoid beliefs in adolescents
Source: JCPP Adv. 2024 Sep 11;5(3):e12280. doi: 10.1002/jcv2.12280 (PMC12446695; doi:10.1002/jcv2.12280)
Supplement: Supplementary file 1 — Supporting Information S1 [file JCV2-5-e12280-s001.docx]

Figure S1

Depiction of all four candidate models for confirmatory factor analysis of paranoia and social anxiety


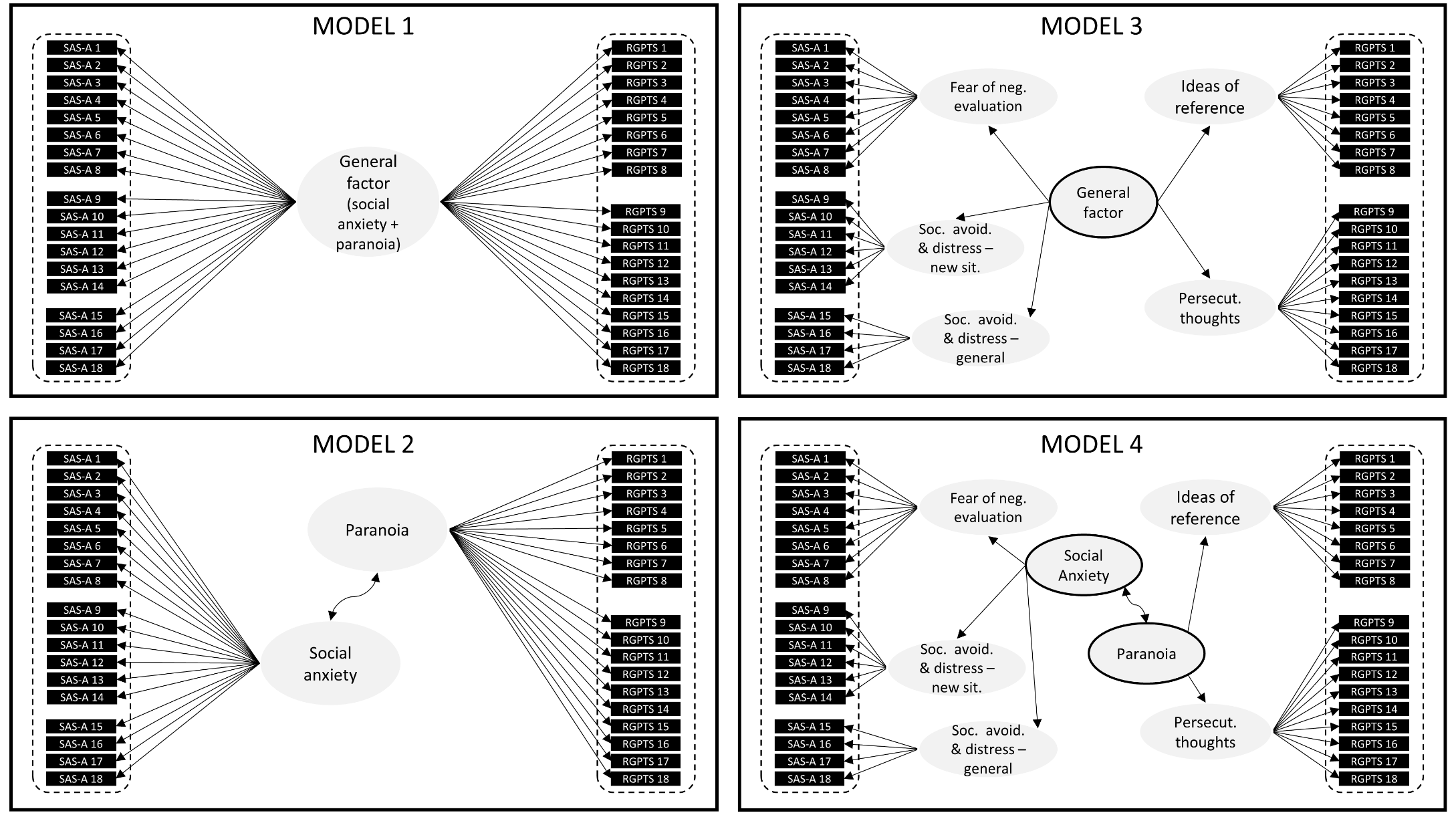


Table S1.

Descriptive statistic and response distribution of all R-GPTS and SAS-A items

|  |  | Descriptive statistics | | | | Distribution of responses | | | | |
| --- | --- | --- | --- | --- | --- | --- | --- | --- | --- | --- |
| # | Item content (abbreviated) | M | SD | skew | kurtosis | 0 | 1 | 2 | 3 | 4 |
|  | R-GPTS |  |  |  |  |  |  |  |  |  |
| 1 | friends gossiping about me | 1.41 | 1.34 | 0.50 | -0.98 | 35.6% | 20.5% | 20.4% | 14.1% | 9.4% |
| 2 | people referring to me | 1.36 | 1.21 | 0.52 | -0.71 | 31.5% | 25.7% | 24.5% | 12.4% | 6.0% |
| 3 | friends/colleagues judge me critically | 1.39 | 1.36 | 0.52 | -1.03 | 37.8% | 19.9% | 17.7% | 15.4% | 9.3% |
| 4 | people laughed behind my back | 1.34 | 1.36 | 0.64 | -0.87 | 37.9% | 23.3% | 15.7% | 12.9% | 10.1% |
| 5 | people avoiding me | 1.06 | 1.26 | 0.95 | -0.27 | 47.7% | 21.2% | 15.2% | 9.6% | 6.3% |
| 6 | people dropping hints | 0.86 | 1.10 | 1.05 | -0.01 | 53.2% | 21.4% | 13.9% | 9.8% | 1.8% |
| 7 | people were not what they seemed. | 1.61 | 1.41 | 0.28 | -1.27 | 31.8% | 18.5% | 18.4% | 19.0% | 12.3% |
| 8 | upset by people talking behind my back | 1.54 | 1.45 | 0.40 | -1.25 | 35.6% | 18.2% | 16.6% | 16.1% | 13.6% |
| 9 | individuals had it in for me | 1.17 | 1.39 | 0.85 | -0.66 | 48.3% | 17.2% | 13.9% | 10.1% | 10.4% |
| 10 | staring to make me to feel threatened | 0.95 | 1.27 | 1.11 | 0.01 | 54.6% | 17.1% | 13.4% | 8.3% | 6.6% |
| 11 | people purposefully annoyed me | 1.57 | 1.40 | 0.34 | -1.22 | 32.3% | 19.9% | 18.1% | 17.9% | 11.9% |
| 12 | conspiracy against me | 0.77 | 1.15 | 1.35 | 0.71 | 61.4% | 14.9% | 12.8% | 7.0% | 4.0% |
| 13 | someone wanted to hurt me | 0.72 | 1.14 | 1.44 | 0.96 | 63.9% | 14.2% | 11.1% | 7.1% | 3.6% |
| 14 | people want to confuse me | 0.73 | 1.09 | 1.38 | 0.91 | 61.4% | 16.1% | 13.4% | 6.1% | 3.0% |
| 15 | being persecuted | 0.67 | 1.11 | 1.55 | 1.27 | 66.7% | 13.1% | 9.8% | 7.1% | 3.3% |
| 16 | people want to make me feel bad | 0.91 | 1.23 | 1.14 | 0.13 | 56.0% | 15.4% | 15.7% | 7.1% | 5.8% |
| 17 | purposeful hostility towards me | 1.07 | 1.32 | 0.93 | -0.47 | 50.3% | 18.5% | 12.1% | 11.8% | 7.3% |
| 18 | anger about intention to hurt me | 0.80 | 1.26 | 1.36 | 0.49 | 64.6% | 11.8% | 8.8% | 8.8% | 6.1% |
|  |  | M | SD | skew | kurtosis | 1 | 2 | 3 | 4 | 5 |
|  | SAS-A |  |  |  |  |  |  |  |  |  |
| 1 | Worrying about what others say | 2.51 | 1.16 | 0.46 | -0.59 | 21.0% | 33.6% | 25.2% | 13.7% | 6.5% |
| 2 | Worrying about not being liked | 2.44 | 1.19 | 0.47 | -0.73 | 25.8% | 30.6% | 22.9% | 14.7% | 6.0% |
| 3 | Worrying about others thoughts | 2.30 | 1.21 | 0.59 | -0.70 | 33.3% | 27.7% | 20.2% | 13.6% | 5.3% |
| 4 | Being afraid of not being liked | 2.48 | 1.23 | 0.47 | -0.84 | 25.2% | 32.3% | 18.5% | 17.1% | 7.0% |
| 5 | Being teased | 2.08 | 1.16 | 0.85 | -0.21 | 41.2% | 27.3% | 17.9% | 9.4% | 4.1% |
| 6 | Not being liked when arguing | 2.07 | 1.17 | 0.88 | -0.22 | 42.2% | 27.0% | 16.6% | 9.9% | 4.3% |
| 7 | Others make fun of me | 2.07 | 1.18 | 0.92 | -0.15 | 41.9% | 28.5% | 14.9% | 10.1% | 4.6% |
| 8 | Peers talking behind my back | 2.13 | 1.20 | 0.81 | -0.39 | 40.9% | 26.0% | 17.4% | 10.8% | 5.0% |
| 9 | Nervous around unknown peers | 2.63 | 1.36 | 0.36 | -1.11 | 26.2% | 26.3% | 18.4% | 16.6% | 12.6% |
| 10 | Shy around unknown people | 2.75 | 1.39 | 0.24 | -1.22 | 24.3% | 23.8% | 19.0% | 18.1% | 14.7% |
| 11 | Nervous around new people | 2.59 | 1.32 | 0.37 | -1.05 | 26.7% | 26.0% | 19.7% | 17.1% | 10.6% |
| 12 | Nervous around certain people | 2.52 | 1.29 | 0.47 | -0.90 | 26.5% | 29.8% | 18.7% | 15.4% | 9.6% |
| 13 | Doing new things in front of others | 2.75 | 1.37 | 0.12 | -1.24 | 26.5% | 17.7% | 22.5% | 21.2% | 12.1% |
| 14 | Only talking to familiar people | 2.66 | 1.35 | 0.30 | -1.13 | 25.7% | 24.7% | 19.9% | 17.6% | 12.3% |
| 15 | Fear of inviting others/being told no | 2.40 | 1.34 | 0.56 | -0.89 | 34.8% | 23.0% | 19.7% | 12.6% | 9.9% |
| 16 | Ask others to do things with me | 2.27 | 1.34 | 0.67 | -0.84 | 41.6% | 20.2% | 16.6% | 13.4% | 8.3% |
| 17 | Quiet in groups | 2.28 | 1.16 | 0.62 | -0.49 | 31.0% | 30.8% | 22.0% | 11.4% | 4.8% |
| 18 | Shy around familiar peers | 1.88 | 1.09 | 1.03 | 0.07 | 51.0% | 22.2% | 16.6% | 8.0% | 2.3% |

Table S2

Standardized item loadings, factor loadings, and co-variances across all four models

| Item/factor | Model 1 | Model 2 | Model 3 | Model 4 |
| --- | --- | --- | --- | --- |
| SAS-A 1 | 0.815 | 0.842 | 0.886 | 0.888 |
| SAS-A 2 | 0.850 | 0.878 | 0.929 | 0.932 |
| SAS-A 3 | 0.840 | 0.872 | 0.914 | 0.917 |
| SAS-A 4 | 0.828 | 0.870 | 0.897 | 0.900 |
| SAS-A 5 | 0.865 | 0.827 | 0.865 | 0.858 |
| SAS-A 6 | 0.835 | 0.830 | 0.854 | 0.850 |
| SAS-A 7 | 0.846 | 0.800 | 0.842 | 0.835 |
| SAS-A 8 | 0.818 | 0.842 | 0.845 | 0.844 |
| SAS-A 9 | 0.696 | 0.802 | 0.903 | 0.901 |
| SAS-A 10 | 0.673 | 0.794 | 0.925 | 0.922 |
| SAS-A 11 | 0.696 | 0.809 | 0.926 | 0.925 |
| SAS-A 12 | 0.777 | 0.860 | 0.885 | 0.889 |
| SAS-A 13 | 0.551 | 0.647 | 0.749 | 0.750 |
| SAS-A 14 | 0.721 | 0.815 | 0.855 | 0.858 |
| SAS-A 15 | 0.754 | 0.829 | 0.883 | 0.888 |
| SAS-A 16 | 0.789 | 0.839 | 0.897 | 0.893 |
| SAS-A 17 | 0.639 | 0.698 | 0.762 | 0.765 |
| SAS-A 18 | 0.644 | 0.669 | 0.728 | 0.724 |
| R-GPTS 1 | 0.762 | 0.897 | 0.847 | 0.841 |
| R-GPTS 2 | 0.694 | 0.762 | 0.781 | 0.784 |
| R-GPTS 3 | 0.762 | 0.728 | 0.858 | 0.856 |
| R-GPTS 4 | 0.790 | 0.839 | 0.866 | 0.871 |
| R-GPTS 5 | 0.800 | 0.808 | 0.837 | 0.838 |
| R-GPTS 6 | 0.673 | 0.740 | 0.743 | 0.753 |
| R-GPTS 7 | 0.683 | 0.708 | 0.759 | 0.756 |
| R-GPTS 8 | 0.761 | 0.786 | 0.848 | 0.843 |
| R-GPTS 9 | 0.747 | 0.824 | 0.821 | 0.825 |
| R-GPTS 10 | 0.721 | 0.815 | 0.839 | 0.840 |
| R-GPTS 11 | 0.707 | 0.766 | 0.731 | 0.740 |
| R-GPTS 12 | 0.729 | 0.825 | 0.845 | 0.846 |
| R-GPTS 13 | 0.655 | 0.785 | 0.841 | 0.838 |
| R-GPTS 14 | 0.745 | 0.806 | 0.818 | 0.818 |
| R-GPTS 15 | 0.701 | 0.793 | 0.837 | 0.833 |
| R-GPTS 16 | 0.787 | 0.848 | 0.858 | 0.858 |
| R-GPTS 17 | 0.758 | 0.851 | 0.872 | 0.873 |
| R-GPTS 18 | 0.698 | 0.792 | 0.849 | 0.844 |
| Factor loadings (Model 3/4) |  |  |  |  |
| Fear of neg. evaluation | - | - | 0.933 | 0.882 |
| Soc. avoidance & distress - new situations | - | - | 0.822 | 0.891 |
| Soc. avoidance & distress - general | - | - | 0.904 | 0.966 |
| Ideas of Refrence | - | - | 0.831 | 0.974 |
| Persecutory thoughts | - | - | 0.769 | 0.879 |
| Covariances (Model 2/4) |  |  |  |  |
| Correlation Paranoia/Social Anxiety |  | 0.747 | - | 0.759 |

Table S3

Results of one way ANOVAs with cut off group (both low, social anxiety high, paranoia high, Both high) as independent variable

| Dependent variable | F(3,600) | p | p _Bonf.-corr._ | η²_partial_ |
| --- | --- | --- | --- | --- |
| General distress (DASS total score) | 155.76 | <0.001 | <0.001 | 0.438 |
| Adverse life events (sum) | 32.45 | <0.001 | <0.001 | 0.140 |
| Loneliness (UCLA) | 160.47 | <0.001 | <0.001 | 0.445 |
| Bullying | 117.76 | <0.001 | <0.001 | 0.371 |
| Discrimination (EDS) | 68.12 | <0.001 | <0.001 | 0.254 |
| School membership | 49.27 | <0.001 | <0.001 | 0.198 |
| Belonging (total) | 21.14 | <0.001 | <0.001 | 0.096 |
| Social comparison (SCS) | 73.68 | <0.001 | <0.001 | 0.269 |
| Avoidant attachment | 39.64 | <0.001 | <0.001 | 0.165 |
| Anxious attachment | 108.45 | <0.001 | <0.001 | 0.352 |

Table S4

Results of post-hoc comparison for between cut-off group differences in all psychosocial variables

| Dependent variable | Cut off_group A | Cut off group B | Mean Difference (A-B) | SE | p_Bonf. corr._ |
| --- | --- | --- | --- | --- | --- |
|  |  |  |  |  |  |
| General distress  DASS total score | Both low | Social anxiety high | -12.34*** | 1.26 | <0.001 |
|  |  | Paranoia high | -9.94*** | 1.75 | <0.001 |
|  |  | Both high | -22.58*** | 1.08 | <0.001 |
|  | Social anxiety high | Paranoia high | 2.40 | 2.01 | ≈1.000 |
|  |  | Both high | -10.24*** | 1.47 | <0.001 |
|  | Paranoia high | Both high | -12.64*** | 1.91 | <0.001 |
| Adverse life events (sum) | Both low | Social anxiety high | -0.51 | 0.23 | 0.187 |
|  |  | Paranoia high | -1.57*** | 0.33 | <0.001 |
|  |  | Both high | -1.87*** | 0.20 | <0.001 |
|  | Social anxiety high | Paranoia high | -1.06* | 0.38 | 0.030 |
|  |  | Both high | -1.37*** | 0.27 | <0.001 |
|  | Paranoia high | Both high | -0.31 | 0.36 | ≈1.000 |
| Loneliness (UCLA) | Both low | Social anxiety high | -6.06*** | 0.47 | <0.001 |
|  |  | Paranoia high | -3.76*** | 0.65 | <0.001 |
|  |  | Both high | -8.08*** | 0.40 | <0.001 |
|  | Social anxiety high | Paranoia high | 2.30* | 0.75 | 0.013 |
|  |  | Both high | -2.02** | 0.55 | 0.001 |
|  | Paranoia high | Both high | -4.32*** | 0.71 | <0.001 |
| Bullying | Both low | Social anxiety high | -1.83*** | 0.39 | <0.001 |
|  |  | Paranoia high | -3.51*** | 0.54 | <0.001 |
|  |  | Both high | -6.12*** | 0.33 | <0.001 |
|  | Social anxiety high | Paranoia high | -1.68* | 0.62 | 0.040 |
|  |  | Both high | -4.29*** | 0.45 | <0.001 |
|  | Paranoia high | Both high | -2.61*** | 0.58 | <0.001 |
| Discrimination (EDS) | Both low | Social anxiety high | -1.86** | 0.56 | 0.006 |
|  |  | Paranoia high | -5.07*** | 0.78 | <0.001 |
|  |  | Both high | -6.55*** | 0.48 | <0.001 |
|  | Social anxiety high | Paranoia high | -3.21** | 0.90 | 0.002 |
|  |  | Both high | -4.69*** | 0.65 | <0.001 |
|  | Paranoia high | Both high | -1.48 | 0.85 | 0.491 |

Table S4 (cont.)

| Dependent variable | Cut off_group A | Cut off group B | Mean Difference (A-B) | SE | p_Bonf. corr_. |
| --- | --- | --- | --- | --- | --- |
| School membership | Both low | Social anxiety high | 9.41*** | 1.54 | <0.001 |
|  |  | Paranoia high | 9.33*** | 2.14 | <0.001 |
|  |  | Both high | 15.04*** | 1.32 | <0.001 |
|  | Social anxiety high | Paranoia high | -0.08 | 2.46 | ≈1.000 |
|  |  | Both high | 5.63* | 1.79 | 0.011 |
|  | Paranoia high | Both high | 5.71 | 2.33 | 0.087 |
| Belonging (total) | Both low | Social anxiety high | 9.97*** | 1.87 | <0.001 |
|  |  | Paranoia high | 5.31 | 2.60 | 0.250 |
|  |  | Both high | 11.12*** | 1.61 | <0.001 |
|  | Social anxiety high | Paranoia high | -4.67 | 2.99 | 0.711 |
|  |  | Both high | 1.15 | 2.18 | ≈1.000 |
|  | Paranoia high | Both high | 5.81 | 2.83 | 0.242 |
| Social comparison (SCS) | Both low | Social anxiety high | 18.15*** | 1.86 | <0.001 |
|  |  | Paranoia high | 4.42 | 2.58 | .526 |
|  |  | Both high | 20.8*** | 1.60 | <0.001 |
|  | Social anxiety high | Paranoia high | -13.73*** | 2.97 | <0.001 |
|  |  | Both high | 2.66 | 2.16 | ≈1.000 |
|  | Paranoia high | Both high | 16.39*** | 2.81 | <0.001 |
| Avoidant attachment | Both low | Social anxiety high | -6.71*** | 0.81 | <0.001 |
|  |  | Paranoia high | -1.52 | 1.12 | ≈1.000 |
|  |  | Both high | -6.03*** | 0.69 | <0.001 |
|  | Social anxiety high | Paranoia high | 5.19*** | 1.29 | <0.001 |
|  |  | Both high | 0.68 | 0.94 | ≈1.000 |
|  | Paranoia high | Both high | -4.50** | 1.22 | 0.001 |
| Anxious attachment | Both low | Social anxiety high | -8.58*** | 0.90 | <0.001 |
|  |  | Paranoia high | -7.25*** | 1.25 | <0.001 |
|  |  | Both high | -13.00*** | 0.77 | <0.001 |
|  | Social anxiety high | Paranoia high | 1.33 | 1.44 | ≈1.000 |
|  |  | Both high | -4.42*** | 1.05 | <0.001 |
|  | Paranoia high | Both high | -5.75*** | 1.36 | <0.001 |

Note. *** - p<0.001; ** - p<0.01; * - p<0.05

Table S5

Descriptive statistics for all measures used in the full sample and all four social anxiety (SA)/paranoia subgroups

|  |  | Full sample | | Low SA/paranoia | | High SA/low paranoia | | Low SA/high paranoia | | High SA/paranoia | |
| --- | --- | --- | --- | --- | --- | --- | --- | --- | --- | --- | --- |
|  | theoretical  range | M | SD | M | SD | M | SD | M | SD | M | SD |
| Ideas of reference (R-GPTS) | 0 - 32 | 10.56 | 8.86 | 5.20 | 5.26 | 11.70 | 5.60 | 18.69 | 4.88 | 22.65 | 4.88 |
| Persecutory thoughts (R-GPTS) | 0 - 40 | 9.38 | 10.50 | 3.11 | 4.18 | 8.26 | 5.67 | 22.59 | 6.65 | 24.04 | 8.21 |
| Social Anxiety (SAS-A Total) | 18 - 90 | 42.82 | 18.48 | 30.65 | 9.13 | 60.68 | 8.92 | 38.74 | 6.98 | 67.02 | 11.04 |
| General distress (DASS total) | 0 - 63 | 13.00 | 13.81 | 6.01 | 7.26 | 18.35 | 11.17 | 15.95 | 11.87 | 28.59 | 15.65 |
| Adverse life events (sum) | 0 - 25 | 1.97 | 2.08 | 1.41 | 1.65 | 1.92 | 1.75 | 2.97 | 2.43 | 3.28 | 2.55 |
| Loneliness (UCLA) | 6 - 24 | 12.60 | 5.17 | 9.86 | 3.75 | 15.92 | 3.93 | 13.62 | 4.23 | 17.94 | 4.00 |
| Bullying | 0 - 25 | 3.43 | 4.01 | 1.69 | 2.15 | 3.52 | 3.07 | 5.21 | 3.30 | 7.81 | 5.14 |
| Discrimination (EDS) | 0 - 25 | 6.52 | 5.34 | 4.59 | 4.40 | 6.45 | 3.52 | 9.67 | 4.98 | 11.15 | 5.66 |
| School membership | 18 - 90 | 55.46 | 14.13 | 60.46 | 12.94 | 51.05 | 10.63 | 51.13 | 10.68 | 45.42 | 13.75 |
| Belonging (total) | 0 - 72 | 39.14 | 16.16 | 43.15 | 15.85 | 33.18 | 13.97 | 37.85 | 12.11 | 32.03 | 15.94 |
| Social comparison (SCS) | 11 - 110 | 65.05 | 17.87 | 72.13 | 14.75 | 53.99 | 12.79 | 67.72 | 15.31 | 51.33 | 18.18 |
| Avoidant attachment | 10 - 50 | 28.82 | 7.27 | 26.55 | 6.75 | 33.26 | 6.04 | 28.08 | 6.36 | 32.58 | 6.85 |
| Anxious attachment | 10 - 50 | 27.28 | 9.18 | 22.96 | 7.75 | 31.54 | 6.77 | 30.21 | 6.45 | 35.95 | 7.08 |

Table S6

Correlation between all measures used in this study

|  |  | 2 | 3 | 4 | 5 | 6 | 7 | 8 | 9 | 10 | 11 | 12 | 13 |
| --- | --- | --- | --- | --- | --- | --- | --- | --- | --- | --- | --- | --- | --- |
| 1 | Ideas of reference (R-GPTS) | 0.82 | 0.71 | 0.65 | 0.38 | 0.68 | 0.62 | 0.55 | -0.46 | -0.3 | -0.46 | 0.38 | 0.66 |
| 2 | Persecutory thoughts (R-GPTS) |  | 0.66 | 0.65 | 0.42 | 0.60 | 0.67 | 0.59 | -0.47 | -0.27 | -0.41 | 0.31 | 0.59 |
| 3 | Social Anxiety (SAS-A Total) |  |  | 0.70 | 0.31 | 0.76 | 0.56 | 0.45 | -0.47 | -0.38 | -0.58 | 0.49 | 0.64 |
| 4 | General distress (DASS total) |  |  |  | 0.45 | 0.69 | 0.58 | 0.56 | -0.52 | -0.36 | -0.48 | 0.41 | 0.61 |
| 5 | Adverse life events (sum) |  |  |  |  | 0.30 | 0.40 | 0.42 | -0.29 | -0.12 | -0.23 | 0.24 | 0.36 |
| 6 | Loneliness (UCLA) |  |  |  |  |  | 0.56 | 0.45 | -0.53 | -0.44 | -0.58 | 0.48 | 0.64 |
| 7 | Bullying |  |  |  |  |  |  | 0.62 | -0.39 | -0.23 | -0.34 | 0.28 | 0.47 |
| 8 | Discrimination (EDS) |  |  |  |  |  |  |  | -0.43 | -0.23 | -0.28 | 0.32 | 0.53 |
| 9 | School membership |  |  |  |  |  |  |  |  | 0.68 | 0.5 | -0.46 | -0.5 |
| 10 | Belonging (total) |  |  |  |  |  |  |  |  |  | 0.46 | -0.43 | -0.32 |
| 11 | Social comparison (SCS) |  |  |  |  |  |  |  |  |  |  | -0.42 | -0.47 |
| 12 | Avoidant attachment |  |  |  |  |  |  |  |  |  |  |  | 0.55 |
| 13 | Anxious attachment |  |  |  |  |  |  |  |  |  |  |  |  |
